# Sample-to-Answer Point-of-Care Blood Lead Level Test

**DOI:** 10.3390/bios16070393

**Published:** 2026-07-21

**Authors:** Rachel L. Warren, Alexander R. Pueschel, Wei W. Yu, Ian M. White

**Affiliations:** 1 Fischell Department of Bioengineering, University of Maryland, College Park, MD 20742, USA; 2 SKM Instruments Inc., Laramie, WY 82055, USA

**Keywords:** blood lead level, point-of-care, anodic stripping, SWASV, electrochemical sensing

## Abstract

Children who are exposed to lead may have extensive health problems, in particular intelligence deficits and developmental delays. Wide-reaching screening programs are essential to identify children in need of remediation and medical intervention. Lead exposure is most problematic in low- and middle-income countries, as well as in underserved populations in wealthier regions of the world. To increase accessibility, it is critical that screening tools are inexpensive, portable, and easy to use. Here we report a low-cost, handheld, sample-to-answer system for the detection of lead in whole blood samples. Our assay simultaneously lyses blood cells, liberates lead from hemoglobin, aggregates proteins and cellular debris, and separates the solubilized lead from the aggregate via a simple filtration device. Using a screen-printed carbon electrode, anodic stripping voltammetry with a low-cost potentiostat, and our sample-to-answer workflow, we achieved a limit of detection of 1.44 μg/dL, which is below the blood lead reference value established by the US Centers for Disease Control and Prevention (3.5 μg/dL). We validated our system using pre-quantified reference samples from lead-exposed animals and demonstrated excellent agreement with our calibration curve, including for samples near the 3.5 μg/dL threshold.

## 1. Introduction

Lead exposure leads to negative physiological outcomes in nearly every system in the body, including the central nervous, the renal, the hematopoietic, the skeletal, the cardiovascular, and the reproductive systems [1]. Children are especially vulnerable to the effects of lead poisoning, which may cause permanent health ailments and developmental delays [2]. In particular, increased blood lead levels (BLLs) in children are associated with deficits in intelligence [3,4] and academic performance [5], as well as attention/hyperactivity-related behavioral issues [6], even at levels well below 10 μg/dL. BLLs in children have also been correlated with hormonal changes [7] that appear to lead to delays in puberty [8], as well as modulated hormone responses to stress [9].

Despite efforts to eliminate lead from the environment, including the regulation of leaded gasoline and paint, and elimination of lead-containing water pipes, exposure continues to be a concern. In the US, data collected from the National Health and Nutrition Examination Survey (NHANES) demonstrated that 2.5% of children aged 0–5 have a BLL higher than 3.5 μg/dL [10]. Those most affected by lead exposure in the US include people of low socioeconomic status, people living in urban housing built before 1978, and immigrant populations [11,12]. Screening children is critical to identify those in need of remediation and medical treatment [13].

At the same time, a high rate of children in low- and middle-income countries (LMICs) show unacceptably high BLLs. The United Nations Children’s Fund (UNICEF) reported approximately 800 million children globally have BLLs at or above 5 μg/dL [14]. In some countries, even the mean BLL among children is above 5 μg/dL. For example, it has been reported that in Nigeria, Serbia, and the Democratic Republic of the Congo, the mean BLL is greater than 7 μg/dL, while in Pakistan, Cameroon, Egypt, and Senegal, the mean BLL is greater than 8 μg/dL [15]. Technologies for BLL screening in children must be designed for deployment in all regions of the world, especially LMICs.

Current BLL testing methods—inductively coupled plasma mass spectroscopy (ICP-MS) [16] and graphite furnace atomic absorption spectroscopy (GFAAS) [17]—offer excellent sensitivity but require a well-equipped central laboratory. Thus, these methods may not be available in many LMICs. Meanwhile, in the US, testing children would require a blood draw at a clinic and an extended wait for the result. This has led to low screening rates. Point-of-care testing is expected to improve screening rates, but currently only one point-of-care test exists on the market—Meridian LeadCare II [18]. Its penetration has been limited, as the instrument and per-test costs are too expensive for screening applications in the US and LMICs. Thus, there is a need for a point-of-care BLL test that is portable, easy to use, has a low instrumentation and per-test cost, and has sufficient performance to accurately detect BLLs down to the US Centers for Disease Control and Prevention (CDC) blood lead reference value (BLRV) of 3.5 μg/dL.

**Figure 1:**
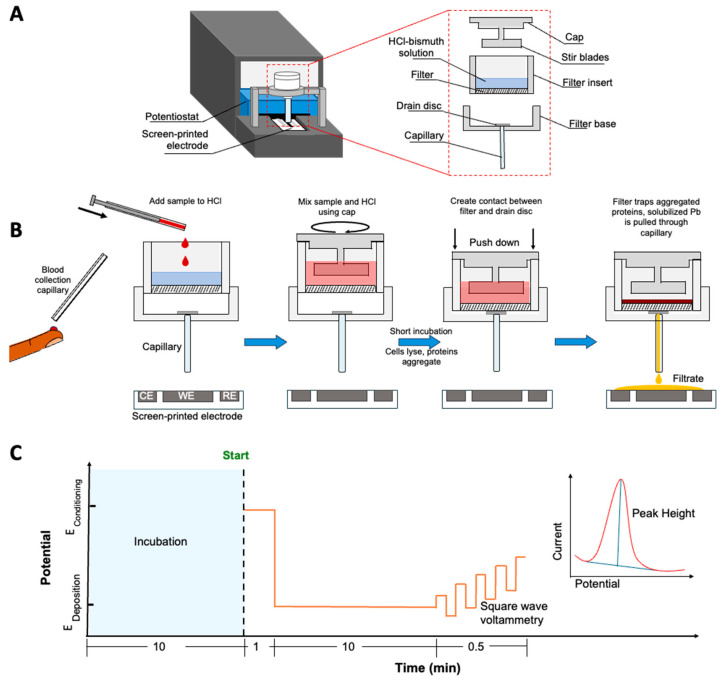
(**A**) The handheld instrument contains a potentiostat and a sample cassette holder. The sample cassette, which is pre-loaded with HCl and bismuth, comprises a filter insert, which has a filter at the bottom, a filter base that has a drain disc and capillary to pull the filtrate from the filter insert, and a cap with fan blades for stirring. (**B**) The whole blood sample (from a finger stick or heel stick) is added into the cassette and stirred with the cap. After a short incubation, the filter insert is pushed into the filter base, contacting the filter with the drain disc. The filtrate drips onto the screen-printed electrode. WE = working electrode, CE = counter electrode, RE = reference electrode. (**C**) Once the filter insert is pushed down, a program is initiated that includes a bismuth–lead co-deposition, then a square wave voltammetry sweep.

Automating the sample preparation and maintaining high performance is challenging, as the lead is bound to hemoglobin in red blood cells [19], implying that the cells must be lysed, the lead must be released from the hemoglobin into solution, and the cell lysate and plasma proteins must be removed to prevent fouling. Here we report a new approach for BLL testing that uses a low-cost handheld instrument, an inexpensive, disposable cassette to filter whole blood, and a low-cost screen-printed electrode for electrochemical detection of lead. Importantly, the test is sample-to-answer and thus can be performed by untrained personnel in the field, such as at a pediatrician’s office or mobile clinic. Our approach is diagrammed in Figure 1. The user loads the screen-printed electrode into the instrument (which contains a low-cost potentiostat) and places the filtering cassette in the instrument above the electrode (Figure 1A). Finger-stick or heel-stick blood collected with a fixed-volume collection capillary is added into the cassette, which contains hydrochloric acid (HCl) and dissolved bismuth. The cap is placed on top of the cassette and spun such that the stir blades in the cap mix the blood and HCl (Figure 1B). The HCl lyses the red blood cells and denatures the hemoglobin, releasing the lead into solution while causing the proteins and cellular debris to aggregate. After a brief incubation, the filter inset is pushed down such that the filter at the bottom of the reservoir contacts the drain disc, which transfers the liquid into the capillary while the filter retains the aggregated proteins and cellular debris. The filtrate falls dropwise from the capillary onto the electrode. After the filter inset has been pushed into place, a program is triggered to perform square wave anodic stripping voltammetry (SWASV) in the potentiostat. In the first step of SWASV, the lead extracted from the blood and the bismuth in the assay reagent are electrochemically co-deposited onto the carbon electrode. The use of bismuth has been shown to substantially enhance the performance of anodic stripping voltammetry for lead detection [20,21]. Following the deposition, a targeted voltage sweep strips the lead from the electrode; the stripping of lead generates a measurable current that is proportional to the amount of lead removed from the electrode. A video of the system operation is provided in the Electronic Supporting Information.

Using this sample-to-answer approach, we achieved a limit of detection of 1.44 μg/dL of lead spiked into 50 μL whole blood. This is below the US CDC BLRV of 3.5 μg/dL. While there is a paucity of literature demonstrating lead detection in whole blood samples, there are a few other reports demonstrating detection of down to ~0.3 mg/dL [22,23,24,25]; however, we believe that ours is the first report of sample-to-answer detection. To further validate our system, we tested whole blood samples from lead-exposed animals with BLLs quantified by reference laboratories and compared the measured values with the calibration curve obtained with the spiked samples. We report excellent agreement, including 10.9% estimation error at 3.5 μg/dL and 1.5% estimation error for 4.0 μg/dL. These results demonstrate that our low-cost portable instrument and sample-to-answer methodology show excellent potential to address the need for point-of-care BLL screening.

## 2. Materials and Methods

### 2.1. Materials

The cassette was assembled with the following materials: Clear Resin V5 3D printing resin (FormLabs, Somerville, MA, USA); 47 mm diameter, 5 μm pore size Polyethersulfone (PES) asymmetric membrane filters (PETEDD9025, Sterlitech, Auburn, WA, USA); 90 mm diameter Polyester (PETE) drain discs (PETEDD9025, Sterlitech); 4 μL end-to-end transfer Microcap© capillaries (Pipette.com); 468MP double-sided adhesive transfer tape (Gizmodorks, Temple City, CA, USA); and Gorilla Clear Glue. Bismuth (III) nitrate pentahydrate (≥98%, ACS grade) was purchased from HiMedia (Kennet Square, PA, USA). HCl (36.5–38%, ACS grade) was purchased from BDH Chemicals (Radnor, PA, USA). A total of 0.32 g of sodium acetate trihydrate was dissolved with 0.144 mL of Acetic Acid (purchased from BDH Chemicals) to a pH of 4.7, as indicated by litmus paper purchased from MQuant (Merck KGaA, Darmstadt, Germany); then DI water was added for a total volume of 50 mL. Certified Pb(II) standards (1000 mg/L in 2% HNO_3_) were obtained from Millipore Sigma (St. Louis, MO, USA). Hyper Value Carbon Sensor (ZPS HYP-000-00150) screen-printed carbon electrodes (SCEs) were purchased from Zimmer & Peacock (Tønsberg, Norway). These sensors include a carbon working electrode, a Ag/AgCl counter electrode, and a Ag/AgCl pseudo-reference electrode. Sheep blood with sodium heparin was purchased from Lampire Biological Laboratories (Pipersville, PA, USA). Reference samples from lead-exposed cows were obtained from the US CDC. Reference samples from lead-exposed goats were obtained from the New York State Health Department. Lead concentration in reference samples had been quantified with ICP-MS.

### 2.2. Disposable Filter Cassette Assembly

**Figure 2:**
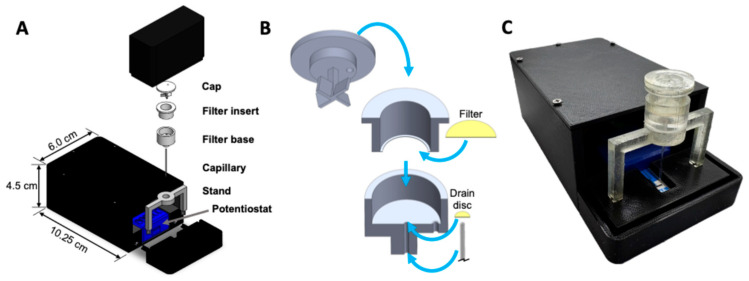
(**A**) Exploded CAD image of the portable device and sample cassette. (**B**) Exploded CAD images of the cap, filter insert, and filter base. (**C**) Photo of the device with the cassette.

The disposable filter cassette is composed of three 3D-printed pieces: a cap with propellor-like stir blades, a sample reservoir with a filter insert, and the base that holds the sample reservoir. The parts are shown in Figure 2A,B. These parts were printed using a FormLabs Form 4 stereolithography printer at 0.025 mm layer thickness. Dimensions for features in the parts in Figure 2B are labeled in Figure S1 and described in detail in the Electronic Supporting Information. The 3D-printed stirring cap has an outer seal that extends to the exact diameter of the filter insert and an inner seal slightly smaller than the initial annular diameter of the filter. The cap includes a cylindrical hole for air replacement of the liquid that drains through the filter. The PES filter (10 mm diameter) is glued in place onto the annular ring of the filter base using clear Gorilla Glue. The bottom of the filter insert is covered with double-sided adhesive transfer tape. The filter base includes a cylindrical hole for friction-fitting the transfer capillary. A second cylindrical hole provides pressure relief to prevent back pressure from building up on the filter when the sample reservoir is pushed down. Experimental results to select optimal filters are explained and presented in Figure S2 in the Electronic Supporting Information.

### 2.3. Handheld Instrument Assembly

The instrument housing was fabricated using a Prusa FDM 3D printer with PLA filament. For device assembly (Figure 2), the SCE is first inserted into the potentiostat contained within the housing. The tray is then positioned with a divot that guides the SCE to the correct insertion angle. A removable stand is attached to the tray, allowing for easy installation and access during operation. The filter base, which is pre-assembled with the capillary and drain disc, is subsequently inserted into the stand. The stand height was designed to ensure that the capillary remains in close proximity to the SCE without making contact, thereby preventing scraping while maintaining effective positioning. Finally, the insert is partially seated into the base, and the cap is secured on top to complete the assembly.

### 2.4. Electrochemistry

Electrochemical measurements were performed with a PalmSens EmStat3 portable potentiostat (PalmSens BV, CL Houten, The Netherlands). SPEs were pre-cleaned with 0.1 M acetate buffer (pH 4.5) using three cyclic voltammetry cycles. Square wave anodic stripping voltammetry (SWASV) was performed with an initial conditioning step of 0.5 V for 1 min followed by a deposition at −1.0 V, during which bismuth and lead were co-deposited onto the electrode surface. Following a 30 s equilibration time, a square wave voltage sweep was conducted from −0.7 V to −0.2 V at a frequency of 20 Hz and a step of 9 mV.

### 2.5. Peak Current Determination

Electrochemical data were processed using a custom R script. To isolate the signal region of interest, current values within a defined potential window of −0.6 V to −0.2 V were extracted at the location of the stripping peak. All possible combinations of left and right boundary points and intermediate peak candidates were evaluated. A linear baseline was constructed between boundary points, and peak height was defined as the maximum vertical deviation of the signal above the baseline. The largest positive deviation was reported as the measured peak current.

### 2.6. Optical Absorbance Measurements

Filtrates from blood samples processed by our device were collected into microtubes below the drain capillaries. Filtrates were then dispensed into a 96-well plate, which was analyzed for absorbance at 500 nm with an Agilent BioTek Synergy LX multimode reader (Agilent, Santa Clara, CA, USA).

### 2.7. Testing Blood Samples with Centrifugation-Based Sample Preparation

Spiked lead samples were prepared by serial dilution with purchased whole blood from sheep. Blood samples were incubated with spiked lead at 4 °C for at least 2 h before being used. A total of 50 µL of blood was added to 75 µL of 1.8 M HCl with 500 ppb bismuth (within the reported range of effective bismuth concentration for effective detection of lead with anodic stripping voltammetry [20]). The blood was mixed with a pipette tip for 30 s then incubated at room temp for 10 min. The samples were centrifuged at 2000× *g* for 2 min. A total of 50 µL of the supernatant was removed and added to the electrode.

### 2.8. Testing Blood Samples with Filtration-Based Sample Preparation

Spiked lead samples were prepared by serial dilution with purchased whole blood from sheep. Blood samples were incubated with spiked lead at 4 °C for at least 2 h before being used. A total of 50 μL of spiked sheep blood was added to the filter insert, which contained 75 μL of HCl/bismuth solution. The mixing cap was added and turned three revolutions. After 10 min, the filter insert was pushed down into the filter base. At that time, the SWASV program was initiated (described above).

The limit of detection (LOD) and limit of quantification (LOQ) were determined as the concentrations that generate a signal that is 3σ_blank_ and 10σ_blank_ above the mean of the blank signal, respectively. The Hill equation (N = 1.6556) was fitted to the measured peak current values from the spiked samples. This equation was used to estimate the lead concentration in the reference samples; the estimate was compared to the value reported by ICP-MS.

## 3. Results and Discussion

### 3.1. Lead Separation Using HCl

**Figure 3:**
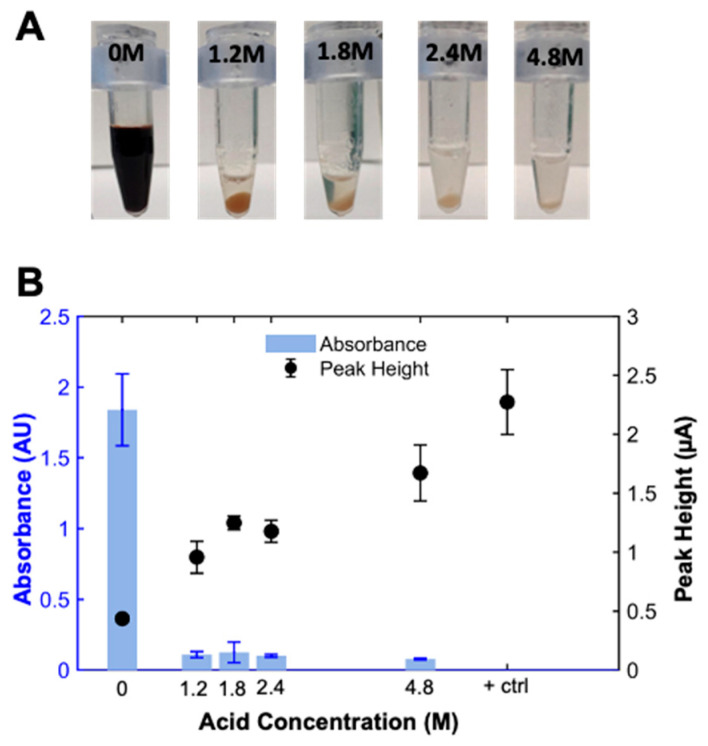
(**A**) Photos of filtrate with varying HCl concentrations added to the sample. (**B**) (Left axis) Optical absorbance values at 500 nm for the filtrates. (Right axis) Measured SWASV peak current value for each filtrate. “+ ctrl” = 25 mg/dL Pb in acetate buffer. Error bars represent +/− one standard deviation for N = 3.

It has been reported that 99% of lead in blood is contained within red blood cells and bound to hemoglobin [19]. Thus, to quantify blood lead levels, it is necessary to lyse the blood cells, denature hemoglobin, solubilize lead, and remove cellular debris and plasma proteins. We hypothesized that HCl would enable all of these processes while limiting the number of user steps. We observed that the addition of HCl to whole blood lysed the cells and rapidly promoted aggregation of proteins and cellular debris, leaving a clear layer in which the lead is dissolved. This would enable the separation of lead from cellular and protein components using a filter. The photos in Figure 3A show filtered blood samples (lead-exposed cow blood samples from the CDC, 25 μg/dL lead) with various concentrations of HCl, while Figure 3B quantifies the filtrate clarity with absorbance measurements (left vertical axis). With no acid (DI water only), the filtered sample is dark in color, demonstrating that a significant amount of hemoglobin passed through the filter (a fraction of the blood cells were lysed due to a freeze–thaw cycle and the addition of DI water). As the HCl concentration is increased, only a small amount of debris passes through the filter. The amount of debris decreases for increasing HCl.

We further hypothesized that the released lead would complex with the chloride ions and thus would be solubilized in the low-pH environment. Figure 3B (right vertical axis) shows the measured SWASV peak current due to lead in the filtrate (blood sample from lead-exposed cow, provided by the US CDC, 25 μg/dL lead) for various HCl concentrations. The measured peak current is near 0 when no HCl is added. The measured peak current increases significantly for 1.2 M HCl, and is higher still for 1.8 M HCl. However, the measured peak current for 2.4 M HCl was not significantly different than that for 1.8 M HCl. While 4.8 M HCl showed lower debris and higher measured peak current, we ultimately selected 1.8 M HCl as a compromise between maximizing the signal and minimizing the safety risk of using a high HCl concentration.

**Figure 4:**
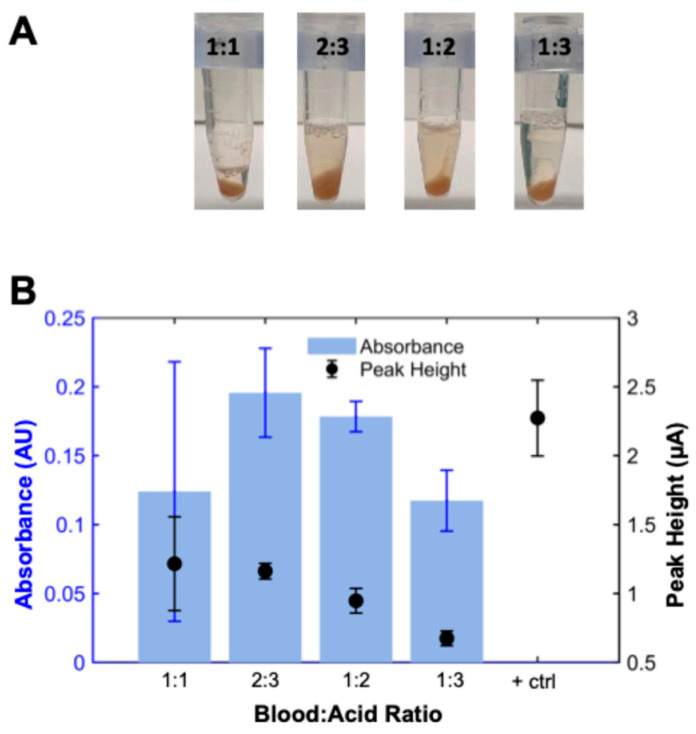
(**A**) Photos of filtrate with varying blood:HCL volume ratios. (**B**) (Left axis) Optical absorbance values at 500 nm for the filtrates. (Right axis) Measured SWASV peak current value for each filtrate. “+ ctrl” = 25 mg/dL Pb in acetate buffer. Error bars represent +/− one standard deviation for N = 3.

We also investigated the optimal ratio of sample volume to HCl volume. A higher blood:HCl ratio implies a higher lead ion concentration in the analyzed volume, but may result in less liquid supernatant volume, which may lead to increased variability in measurements. The photos in Figure 4A show the amount of liquid (including debris) that passed through the filter for various blood:HCl volume ratios. For a 1:1 ratio, only about 10% of the initial volume is recovered through the filter. For a 2:3 ratio and lower, the recovered volume increased to greater than 40% of the initial volume. The peak currents for SWASV (blood sample from lead-exposed cow, provided by the US CDC, 25 μg/dL lead) are plotted in Figure 4B. The 2:3 volume ratio proved to be the optimum ratio. Thus, in the remaining experiments, we used a 2:3 ratio of blood to 1.8 M HCl.

### 3.2. Optimization of Pre-Filtering Conditions

**Figure 5:**
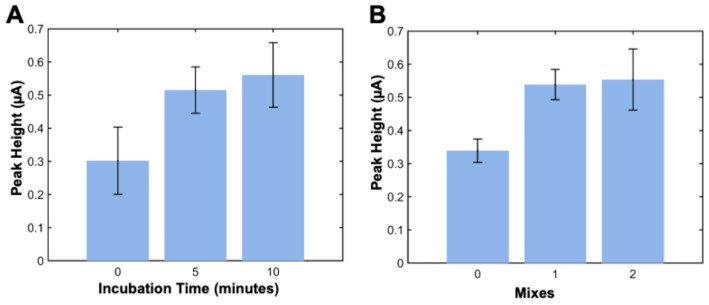
(**A**) Measured SWASV peak current value for varying blood–HCl incubation times. (**B**) Measured SWASV peak current value for varying mixing protocols: no mixes; one mix prior to 10 min incubation; and one mix prior to incubation and a second mix 5 min after loading, prior to an additional 5 min incubation. Error bars represent +/− one standard deviation for N = 3.

In addition to the reagent combinations, we also optimized the procedure for blood sample pre-treatment. First, we investigated the dependence of measured peak current values on blood:HCl incubation time before the sample passes through the filter. Additional incubation time is expected to result in higher release of lead from hemoglobin, but it is preferred to minimize the assay time for patient convenience. The SWASV peak current values for 0, 5 min, and 10 min incubation are plotted in Figure 5A. Even for 0 min incubation (i.e., as soon as the blood is added to the HCl in the cassette, the sample is briefly mixed and the filter insert is pressed against the drain disc, causing the sample to pass through the filter), there is a measurable current from lead in the sample (blood sample from lead-exposed cow, provided by the US CDC, 15.9 μg/dL lead). Including a 5 min incubation step increased the SWASV peak current by about 70%, while a 10 min incubation step nearly doubled the SWASV peak current. For all additional measurements, we used a 10 min incubation step, though we note that a 5 min incubation step could be considered, as there is a relatively small difference between the two, but decreasing the assay by 5 min may be of significant value.

Finally, we investigated the benefit of mixing the blood and HCl before the filtering step. We hypothesized that additional mixing would improve the release of lead from the hemoglobin and promote protein aggregation, but we also aim to minimize user steps. Figure 5B shows the SWASV peak current values (blood sample from lead-exposed cow, provided by the US CDC, 15.9 μg/dL) for zero mixing events, one mixing event immediately after the blood is added, and two mixing events (one after the blood is added, one additional mix five minutes later), with a mixing event defined as three revolutions of the cap. The data demonstrates that it is valuable for the user to mix the sample (by spinning the cap when attaching it), and that the additional mixes do not improve performance.

### 3.3. Performance Assessment with Spiked Blood Samples

**Figure 6:**
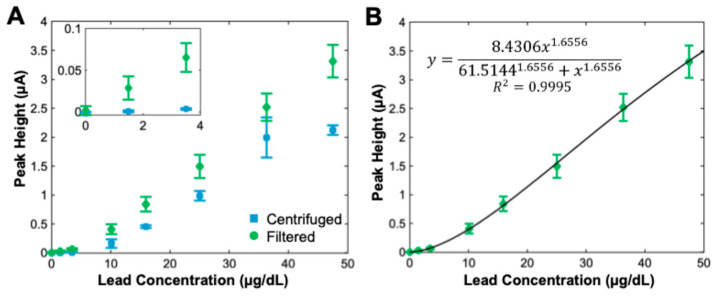
(**A**) Measured SWASV peak current values for various lead concentrations spiked into whole blood samples. Squares represent the use of a centrifuge to remove the aggregated blood components and circles represent the use of the handheld instrument and filtering cassette. (**B**) Data points from the “filtered” experiment in (**A**) fit with a Hill equation. Error bars represent +/− one standard deviation for N = 5.

After optimizing the reagents and protocol, we characterized the performance of our sample-to-answer BLL detection system using spiked whole blood samples. The SWASV peak current values are plotted for a range of spiked lead levels in Figure 6 (selected voltammograms are included in the Supporting Information, Figure S3). For comparison, we also prepared samples by spiking blood with lead, adding HCl and bismuth, and centrifuging the samples to separate the lead from the blood (the supernatant was manually added to the electrodes after centrifugation). As Figure 6 demonstrates, the SWASV peak current was slightly higher for our sample-to-answer system than for the centrifugation. We believe that the slight improvement results from the dripping of the filtrate onto the electrode, as each drop results in convective redistribution of the bismuth and lead, which overcomes the diffusion-limited deposition at the electrode surface (for the centrifuged sample, no convection occurred during deposition after the supernatant was applied to the electrode). While the sensitivity is higher for our sample-to-answer system, we acknowledge that the error bars are larger than for centrifugation-based sample preparation. This is likely due to variations in our system, such as droplet size and filtrate volume.

In Figure 6B we present a calculated Hill fit (N = 1.6556) for the sample-to-answer data set. A Hill fit is expected due to the cooperative nucleation at the Bi-film electrode as well as the saturation of the electrode at higher Pb concentrations. Using the definition for the limit of detection (LoD) as the concentration that would generate a signal that is three times larger than the standard deviation of the mean of the blank sample, we calculated the LoD to be 1.44 μg/dL. This is below the US CDC’s blood level reference value of 3.5 μg/dL, and is thus sufficient for screening applications. We determined the limit of quantification (LoQ) to be 2.87 μg/dL, where the LoQ is the concentration that would generate a signal that is ten times larger than the standard deviation of the mean of the blank sample.

### 3.4. Validation with Reference Whole Blood Samples

**Figure 7:**
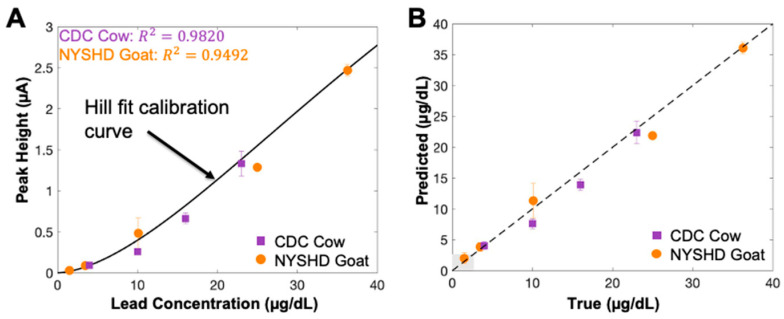
(**A**) Measured SWASV peak current values for various reference whole blood samples from cows and goats exposed to lead, along with the calibration curve from Figure 6. (**B**) Correlation of the predicted blood lead concentration (using the calibration curve) with the actual concentration provided by the reference lab. The shaded region indicates the LoQ. Error bars represent +/− one standard deviation for N = 3.

To validate our sample-to-answer system, we utilized samples from two reference labs (samples from lead-exposed cows from the CDC and samples from lead-exposed goats from the New York State Health Department). The BLL for each reference sample was determined by ICP-MS by the reference labs. In Figure 7A, we plot the measured SWASV peak currents for each sample along with the calibration curve from Figure 6. The estimated lead concentration using SWASV was determined by inserting the measured peak current into the Hill equation (Figure 6B) and calculating the concentration. In Figure 7B, we show the agreement between the spiked calibration samples and the reference samples, where “True” represents the values measured by ICP-MS and “Predicted” represents the value we determined by estimating the concentration from our measured values using the calibration curve. The graph demonstrates excellent agreement between the true and predicted concentration, validating that our proposed sample-to-answer system can accurately quantify blood lead levels using a calibration curve. All estimation errors are listed in Table 1.

## 4. Conclusions

In this study, we demonstrated sample-to-answer detection of lead directly from whole blood samples. To separate the lead from the red blood cells, we added HCl to lyse the red blood cells and denature hemoglobin, thus releasing lead into the acid solution while aggregating the denatured proteins and cellular debris. A simple filtering cassette delivered the solubilized lead to a screen-printed carbon electrode while the filter retained the debris. The entire assay requires only about 20 min, and the time has the potential to be further shortened. The assay is fully automated in a handheld instrument that contains a low-cost potentiostat and positions the filtering cassette over the screen-printed carbon electrode, enabling hands-free filtrate delivery. Using this instrument, we achieved a limit of detection of 1.44 μg/dL of lead spiked into whole blood, safely below the US CDC blood level reference value. After creating a calibration curve from the spiked blood samples, we tested reference whole blood samples from lead-exposed animals. We achieved excellent agreement, including a 10.9% estimation error for 3.5 μg/dL and a 1.5% estimation error for 4.0 μg/dL. Notably, this high performance is achieved despite the use of a low-cost screen-printed carbon electrode and a portable potentiostat.

We anticipate that our sample-to-answer blood lead level test can address the critical need for point-of-care screening throughout the world. The handheld instrument and hands-free operation imply that the test can be performed in the field. Additionally, the instrument can be easily manufactured and consists of relatively low-cost components (estimated cost of goods is included in the Supporting Information, Table S1); the most expensive element is the potentiostat, which could be purchased for ~$200. The cassette components can be mass-produced by injection molding and easily assembled with the filters.

One key opportunity for improvement is to decrease the size of the instrument. While it is currently small enough to be handheld, we expect the components can be redesigned to shrink the volume by 50%. In addition, we expect that further protocol optimization would enable the assay time to be decreased down to 10 min without a significant sensitivity penalty. Finally, we expect that the dependence on a laptop can be eliminated with an onboard microcontroller and simple display without significantly increasing the cost. After optimizations are complete, we believe the system would be ready for field trials with patient samples.

Additionally, we envision that the platform could be extended for the multiplexed detection of heavy metals. Anastasiadou et al. have previously demonstrated that SWASV with bismuth electrodes generates highly specific anodic peaks for various metals, including zinc, cadmium, lead, and copper [26]. This lack of interference between different metals leads to highly specific detection of individual metals as well as quantitative multiplexed detection.

## Figures and Tables

**Table 1:** Comparison between estimated blood lead concentration (using Hill fit calibration curve) and actual blood lead concentration provided by the reference labs for all samples tested. CDC = US Centers for Disease Control and Prevention, NYSHD = New York State Health Department.

Sample	Lead Conc. (µg/dL) by ICP-MS	Estimated Conc. by Sample-to-Answer System	Absolute Estimation Error (µg/dL)	% Estimation Error
#1–CDC Cow	1.49	2.00	0.51	34.13
#2–CDC Cow	3.5	3.88	0.38	10.87
#3–CDC Cow	10.1	11.35	1.25	12.34
#4–CDC Cow	25	21.86	3.14	12.57
#5–CDC Cow	36.3	36.10	0.20	0.55
#7–NYSHD Goat	4	4.06	0.06	1.49
#8–NYSHD Goat	10	7.61	2.39	23.91
#9–NYSHD Goat	16	13.92	2.08	12.97
#10–NYSHD Goat	23	22.37	0.63	2.73

## Data Availability

The data presented in this study are available on request from the corresponding author.

## Supplementary Materials

The following supporting information can be downloaded at: https://www.mdpi.com/article/10.3390/bios16070393/s1. Figure S1: Exploded CAD images of the components of the sample cassette with dimensions labeled; Figure S2: Optimization of filter cassette physical properties consisting of filter pore size or drain disc diameter; Video S1: Video showing the operation of the filtering cassette; Figure S3: Selected voltammograms from the data collected for Figure 6; Table S1: Estimated cost of goods for the cassette.
