# Supplementary material for: Sample-to-Answer Point-of-Care Blood Lead Level Test
Source: Biosensors (Basel). 2026 Jul 21;16(7):393. doi: 10.3390/bios16070393 (PMC13407230; doi:10.3390/bios16070393)
Supplement: Supplementary file 1 [file biosensors-16-00393-s001.zip › S2A blood lead test Supporting Information.pdf]

Sample-to-answer point-of-care blood lead level test

Electronic Supporting Information

Rachel L. Warren[1,†], Alexander R. Pueschel[1,†], Wei W. Yu[2], and Ian M. White[1,*]

1 Fischell Department of Bioengineering, University of Maryland, College Park 20742, United States
2 SKM Instruments Inc, Laramie, WY, USA
† Authors contributed equally
* Correspondence: ianwhite@umd.edu

Disposable filter cassette assembly

Dimensions for the key parts of the filter cassette assembly are shown in Figure S1. The filter holder is an annular cylinder with an outer diameter of 13.23 mm and an inner diameter of 10 mm. A lip at the bottom creates a 2nd annular radius of 4.5 mm with the lip having a thickness of 0.05 mm and length of 0.5 mm. The filter holder's lip is designed to hold 10 mm diameter pieces cut from the 47 mm diameter 5 µm pore size PES asymmetric membrane filters. The 5 µm PES filters are glued in place using clear Gorilla Glue. The bottom of the filter insert is covered with double sided adhesive transfer tape (8″ x 8″) that are cut to the dimensions of the filter insert. The filter base has a 0.934 mm cylindrical hole made during printing for friction fitting the 4 µL end-to-end transfer capillaries. At the bottom of the filter base and on top of the capillary sits a 3 mm piece of PETE drain disc cut from 90 mm PETE drain discs. The filter base is composed of a 3D-printed cylinder with an inner diameter of 13.5 mm and an outer diameter of 16.3 mm forming an anulus with a height of 7 mm. At the bottom of the anulus are two holes extending through the filter base. One is for the capillary (described above); the other sits off center from the 1st hole at a center-to-center distance of 5.75 mm with a diameter of 1 mm and provides pressure relief to prevent back pressure from building up on the filter when the sample reservoir is pushed down. The 3D-printed stirring cap has an outer seal that extends to the exact diameter of the filter insert and an inner seal slightly smaller than the initial annular diameter of the filter. A square central pillar extends with a height of 6.95 mm. Each pillar face has an equally sized fin centered on the face and tangent to the bottom edge, extending from the bottom edge by 3.5 mm. The fins are parallelograms with a base of 0.75 mm and a height of 3 mm.

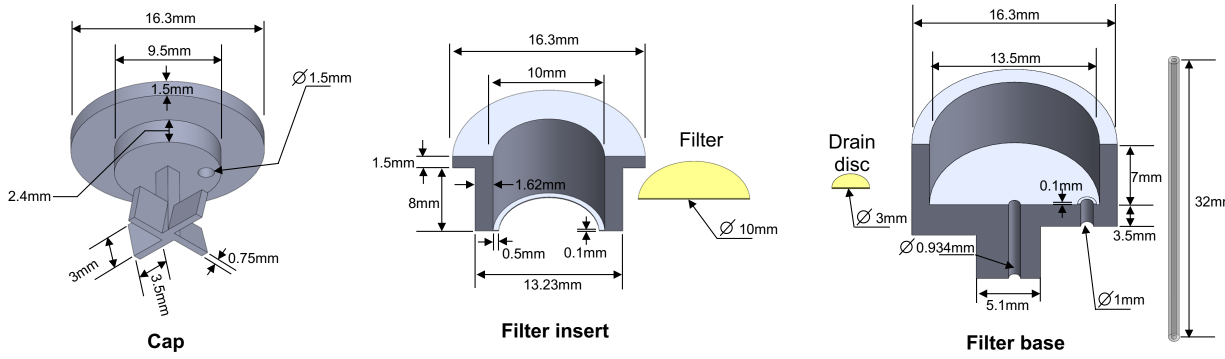

Figure S1. Dimensions of filter cassette components.

Filtration performance with varying pore size and drain disc diameter

We investigated a range of filter pore sizes to determine if there was an optimal size. All membranes tested (1.2 µm, 3 µm, 5 µm) generated a clear filtrate of similar volume (Figure S2(a)). In addition, we investigated a range of drain disc sizes (3 mm, 5 mm, 8 mm). All sizes resulted in similar output (Figure S2(b)): clear filtrate and a filtrate volume that is 50-60% of the sample volume.

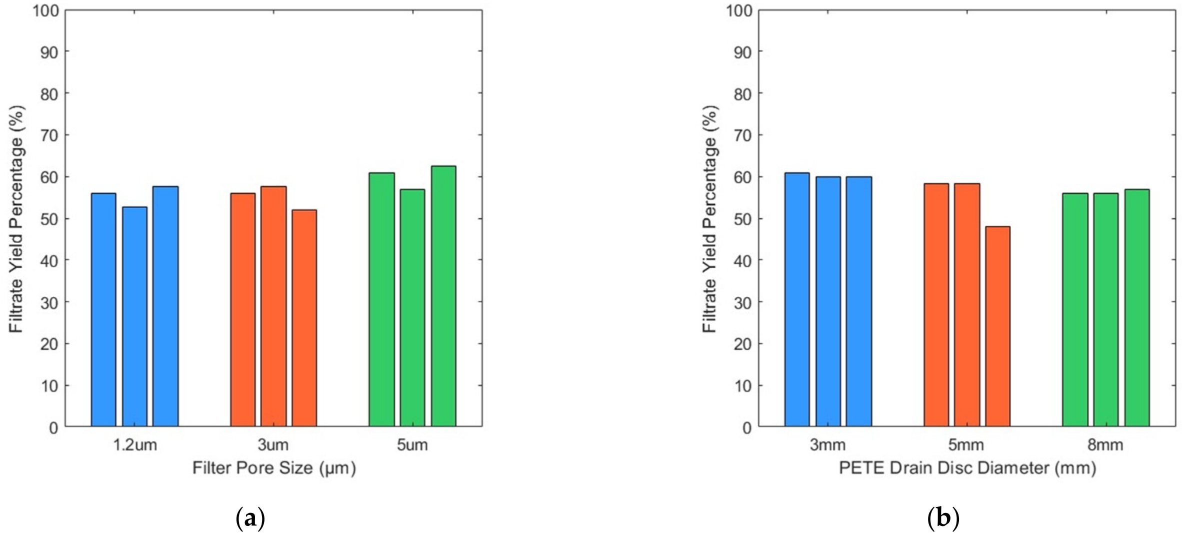

Figure S2. (**a**) Filtrate yield when the filter cassette uses either 1.2, 3, or 5 µm pore size (sheep blood, N = 3); (**b**) Filtrate yield when the filter cassette uses either 3, 5, or 8 mm drain disc diameter (sheep blood, N = 3).

Video showing the operation of the filtering cassette

The provided video shows the operation of the filtering cassette. First, the HCl/bismuth solution is added to the sample reservoir (this could be preloaded during real-world use). The sheep blood sample is then added, followed by the addition of the propellor cap, which is turned three times for one complete mixing event. A 10-minute incubation follows. After this, the filter insert is pressed down, causing the filter and drain disc to be in contact. This forms a liquid connection, allowing the transfer of the filtrate to the capillary, which then drips onto the screen-printed electrode. As the sample drips onto the electrode, the SWASV program is initiated.

Voltammograms for the detection of Pb in blood

Figure S3 shows voltammograms recorded for 0, 3.5 µg/dL, and 10 µg/dL Pb spiked in blood, corresponding to data plotted in Figure 6 (N = 5 for each concentration). The yellow circle marker indicates the peak identified by the software that determines the peak height.

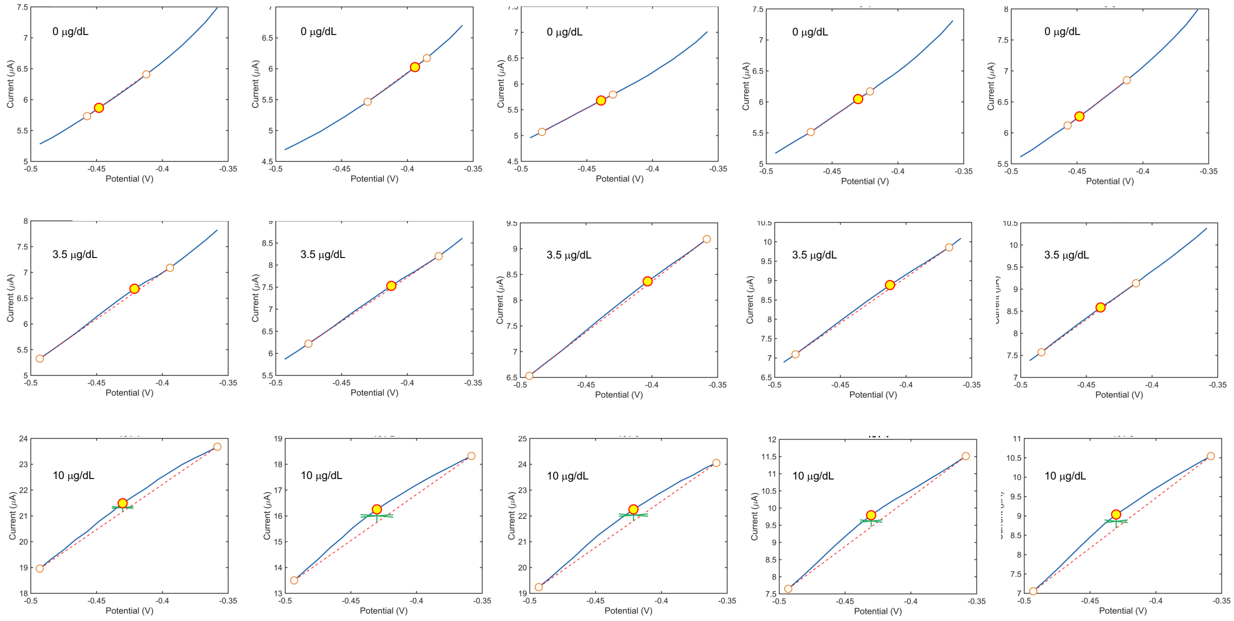

Figure S3. Voltammograms for 0, 3.5 μg/dL, and 10 μg/dL (N = 5) Pb spiked into whole blood. The graphs are displaying the range in which the Pb current peak is detected.

Cost of components

Table S1 provides estimated costs for the components used to assemble the cassette, as well as the cost of the screen-printed carbon electrode.

Table S1. Estimated cost of consumables

**Component**	**Estimated cost (assuming 10,000 units)**
Mixing cap	$0.48
Filter insert	$0.48
Filter base	$0.49
Filter	$0.74
Drain disc	<$0.01
Tape	<$0.01
Capillary	$0.13
Screen-printed carbon electrode	$0.93
**Total cost of goods per test**	**$3.25**
